# 

**Published:** 1886-09

**Authors:** 


### SPECIAL.

We wish to express our thankfulness for the very many flattering notices we have received from the press since the JOURNAL entered upon its more liberal policy. Our crowded columns do not admit of their reproduction, even in a condensed form, and to make selections where all have expressed friendly and encouraging sentiments, would be in questionable taste. To one and all we now tender our acknowledgments, and the hearty good wishes of the JOURNAL for their success in all good works. Note change of office to 206 Broadway.

